# KLF15 Loss-of-Function Mutation Underlying Atrial Fibrillation as well as Ventricular Arrhythmias and Cardiomyopathy

**DOI:** 10.3390/genes12030408

**Published:** 2021-03-12

**Authors:** Ning Li, Ying-Jia Xu, Hong-Yu Shi, Chen-Xi Yang, Yu-Han Guo, Ruo-Gu Li, Xing-Biao Qiu, Yi-Qing Yang, Min Zhang

**Affiliations:** 1 Department of Cardiology, Shanghai Chest Hospital, Shanghai Jiao Tong University, Shanghai 200030, China; frankleelx@163.com (N.L.); shi_hongyu68@sina.com (H.-Y.S.); liruogu@hotmail.com (R.-G.L.); qiuxingbiao@hotmail.com (X.-B.Q.); 2 Department of Cardiology, Shanghai Fifth People′s Hospital, Fudan University, Shanghai 200240, China; xuyingjia@5thhospital.com (Y.-J.X.); 13472626672@163.com (C.-X.Y.); dangochan@126.com (Y.-H.G.); 3 Cardiovascular Research Laboratory, Shanghai Fifth People′s Hospital, Fudan University, Shanghai 200240, China; 4 Center Laboratory, Shanghai Fifth People’s Hospital, Fudan University, 801 Heqing Road, Shanghai 200240, China

**Keywords:** arrhythmia, atrial fibrillation, cardiomyopathy, genetics, transcription factor, KLF15

## Abstract

Atrial fibrillation (AF) represents the most common type of clinical cardiac arrhythmia and substantially increases the risks of cerebral stroke, heart failure and death. Accumulating evidence has convincingly demonstrated the strong genetic basis of AF, and an increasing number of pathogenic variations in over 50 genes have been causally linked to AF. Nevertheless, AF is of pronounced genetic heterogeneity, and the genetic determinants underpinning AF in most patients remain obscure. In the current investigation, a Chinese pedigree with AF as well as ventricular arrhythmias and hypertrophic cardiomyopathy was recruited. Whole exome sequencing and bioinformatic analysis of the available family members were conducted, and a novel heterozygous variation in the KLF15 gene (encoding Krüppel-like factor 15, a transcription factor critical for cardiac electrophysiology and structural remodeling), NM_014079.4: c.685A>T; p.(Lys229*), was identified. The variation was verified by Sanger sequencing and segregated with autosomal dominant AF in the family with complete penetrance. The variation was absent from 300 unrelated healthy subjects used as controls. In functional assays using a dual-luciferase assay system, mutant KLF15 showed neither transcriptional activation of the KChIP2 promoter nor transcriptional inhibition of the CTGF promoter, alone or in the presence of TGFB1, a key player in the pathogenesis of arrhythmias and cardiomyopathies. The findings indicate KLF15 as a new causative gene responsible for AF as well as ventricular arrhythmias and hypertrophic cardiomyopathy, and they provide novel insight into the molecular mechanisms underlying cardiac arrhythmias and hypertrophic cardiomyopathy.

## 1. Introduction

Atrial fibrillation (AF) represents the most frequent type of clinical cardiac dysrhythmia in humans, with an estimated prevalence of 1% for AF in the general population [1]. AF confers a substantially increased risk of ischemic cerebral stroke or other systemic embolism [2,3], cognitive impairment or dementia [4,5,6], tachycardia-induced cardiomyopathy [7], cardiac dysfunction or congestive heart failure [8], ventricular arrhythmias [9], and death [10,11,12]. Hence, AF has become a growing socioeconomic burden [13]. Despite this clinical significance, the molecular pathogenesis underpinning AF remains largely obscure.

Previous epidemiological studies have indicated that structural cardiac diseases and systemic conditions as well as other risk factors, encompassing coronary heart disease, primary hypertension, diabetes mellitus, obstructive sleep apnea and exposure to toxicants, have a role in the development of AF [14,15]. However, accumulating compelling evidence underscores the genetic basis of AF [16,17,18]. Using genome-wide scans with polymorphic genetic markers in AF families and linkage analysis, Brugada and colleagues [19] mapped the first genetic locus for AF on chromosome 10q22–24, although the AF-causing gene has not yet been discovered. By positional candidate gene analysis in a large family with AF, Chen and coworkers [20] located a novel genetic locus for AF on chromosome 11p15.5, and within this chromosomal region identified the first AF-causative gene, Ser140Gly-mutant KCNQ1. To date, in addition to chromosomal duplications/deletions, pathogenic mutations in over 50 genes have been discovered to cause AF, including those encoding ion channels (pore-forming α and auxiliary β subunits), gap junction channels, myocardial structural proteins, cellular signal molecules and cardiac transcription factors [16,17,18,21,22,23,24,25,26,27,28,29,30,31,32,33]. Nevertheless, AF is of substantial genetic heterogeneity, and in the overwhelming majority of patients the genetic components responsible for AF are still elusive.

## 2. Materials and Methods

### 2.1. Study Subjects

**Figure 1:**
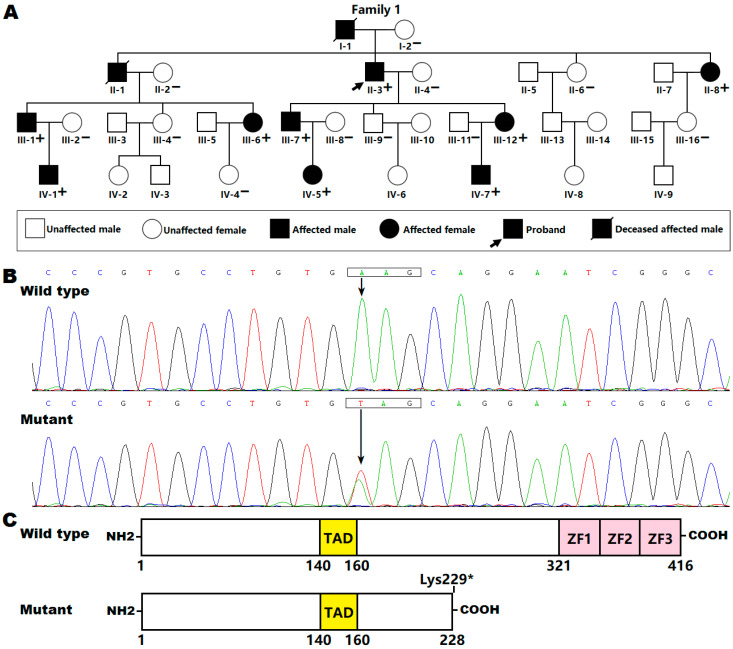
A novel KLF15 mutation responsible for familial atrial fibrillation. (**A**) Pedigree structure for the Chinese family with atrial fibrillation. The Chinese family was arbitrarily designated as family 1. Family members are identified by Roman-Arabic numerals. “+” indicates an individual carrying the heterozygous mutation; “–”, an individual with no mutation. (**B**) Sequence chromatograms showing the heterozygous *KLF15* mutation and its wild-type control. An arrow symbol points to the heterozygous nucleotides of T/A in the proband (mutant) or the homozygous nucleotides of A/A in an unaffected individual (wild type). A rectangle marks 3 nucleotides comprising a codon of *KLF15*. (**C**) Schematic diagrams of the structural domains of human KLF15 proteins displaying the mutation contributing to atrial fibrillation. COOH, carboxyl-terminus; NH2, amino-terminus; TAD, transcriptional activation domain; ZF, zinc finger.

In the present study, a large Chinese family affected with AF was identified (for pedigree see Figure 1A). The available family members were enrolled and clinically evaluated, including a comprehensive review of personal and medical histories, thorough physical examination, standard 12-lead electrocardiogram, echocardiogram and routine laboratory tests. A 48 h ambulatory electrocardiogram and clinical electrophysiological examination were performed when indicated. For the family members who had died, their surviving close relatives were questioned and their hospital records were reviewed. AF was diagnosed and classified according to the 2014 AHA/ACC/HRS guidelines for the management of patients with AF [13]. Additionally, 300 unrelated healthy subjects, who were matched for ethnicity and gender, were recruited as controls. The control subjects were identified from individuals who took an annual physical examination in local hospitals and were enlisted if they had neither electrocardiographic abnormalities nor medical history of AF. All the study participants were enlisted from the Chinese Han population. Clinical data and peripheral venous blood samples were collected from all study subjects. The research complied with all the relevant national regulations and institutional policies, was in accordance with the tenets of the Helsinki Declaration and was approved by the Medical Ethics Committee of the Shanghai Chest Hospital, Shanghai Jiao tong University, China (ethical approval number: KS1101). Written informed consent was provided by study participants or their legal guardians prior to investigation.

### 2.2. Genetic Analysis

Extraction of genomic DNA from each individual’s blood leukocytes was performed using a Wizard Genomic DNA Purification Kit (Promega, Madison, WI, USA). For each study participant, an exome library was constructed with 1 μg of genomic DNA, and captured by utilizing a SureSelectXT Human All Exon V6 Kit (Agilent Technologies, Santa Clara, CA, USA) following the manufacturer’s protocol. Exome libraries were enriched and sequenced on the Illumina HiSeq 2000 Genome Analyzer (Illumina, San Diego, CA, USA), using a HiSeq Sequencing Kit (Illumina) according to the manufacturer’s instructions. Bioinformatic analyses were made as described previously [34,35,36,37]. Based on the possible inheritance models of AF in the pedigree (Figure 1A), the variants that did not match any reasonable inheritance pattern of AF were filtered out. The variants passing the pedigree analysis were annotated using ANNOVAR. A candidate pathogenic variant identified by whole exome sequencing (WES) was confirmed by Sanger sequencing and co-segregation of the variant with AF in the family (Figure 1A). To confirm the variant as deleterious, 300 control individuals were scanned, and population genetics databases such as the Exome Aggregation Consortium (ExAC) database (http://exac.broadinstitute.org/), the Genome Aggregation Database (gnomAD; http://exac.broadinstitute.org/) and the Single Nucleotide Polymorphism database (dbSNP; https://www.ncbi.nlm.nih.gov/snp/) were searched (the firstly accessed date for these links were 16 March 2018) to exclude the possibility of a benign polymorphism and to validate the variant’s novelty.

### 2.3. Expression Plasmids and Site-Directed Mutagenesis

Isolation of total RNA from human heart tissue specimens (previously collected from the discarded cardiac muscle tissues of the patients affected with tetralogy of Fallot undergoing routine cardiac surgery and stored at −80 °C in our laboratory) and preparation of cDNA were performed as described previously [33]. A 1340 bp fragment from nucleotide 151 to 1490 of the human Krüppel-like factor 15 (KLF15) gene (GenBank accession No. NM_014079.4) containing the entire coding sequences of KLF15 was amplified from cDNA by polymerase chain reaction (PCR) using PfuUltra High-Fidelity DNA Polymerase (Stratagene, Santa Clara, CA, USA) and a specific pair of primers (forward primer: 5′-TCTGAATTCGTCCGGCGTGCGCCAAGTTC-3′; reverse primer: 5′-AGAGCGGCCGCTGGGGATCCGGGGTGACGGA-3′). The resultant KLF15 cDNA was doubly digested with restriction enzymes EcoRI and NotI, and then inserted into EcoRI-NotI sites in a pcDNA3.1 plasmid (Invitrogen, Carlsbad, CA, USA) to construct the eukaryotic gene expression plasmid KLF15-pcDNA3.1. The Lys229*-mutant KLF15-pcDNA3.1 plasmid was generated by PCR-based site-directed mutagenesis of wild-type KLF15-pcDNA3.1 using a QuickChange II XL Site-Directed Mutagenesis Kit (Stratagene) with a complementary pair of primers (forward primer: 5′-CAGCCCGTGCCTGTGTAGCAGGAATCGGGCA-3′; reverse primer: 5′-TGCCCGATTCCTGCTACACAGGCACGGGCTG-3′), and was confirmed by PCR sequencing. Similarly, the full-length cDNA of the human transforming growth factor β1 (TGFB1) gene (GenBank accession no. NM_000660.7) was amplified by PCR with DNA polymerase and a specific pair of primers (forward primer: 5′-GTTGAATTCCCAGCCCTGTTCGCGCTCTC-3′; reverse primer: 5′- GTTGCGGCCGCGGGGCGGGGCGGGGCGGGAC-3′), digested with EcoRI and NotI, and then inserted into a pcDNA3.1 plasmid to construct the recombinant expression plasmid TGFB1-pcDNA3.1. A 1701 bp DNA fragment (from ‒1469 to +232, with the transcriptional start site numbered as +1) of the human voltage-gated potassium (Kv) channel-interacting protein 2 (KChIP2) gene (GenBank accession No. NC_000010.11), which harbors multiple consensus Krüppel-binding sites, C(A/T)CCC [38], was amplified from human genomic DNA by PCR with a specific pair of primers (forward primer: 5′-GCCGGTACCTAGGGCATGGGGCACTAAGC-3′; reverse primer: 5′-GCCAAGCTTGGCCCCCGGCGCCCCGCTCC-3′), digested with restriction enzymes (KpnI and HindIII), and inserted into the basic vector pGL4.10 (Promega) to construct a KChIP2 promoter-driven firefly luciferase reporter plasmid (KChIP2-luc). The firefly luciferase reporter plasmid driven by the promoter of connective tissue growth factor (CTGF) gene (CTGF-luc) was constructed as described elsewhere [39].

### 2.4. Cell Culture, Transfection and Dual-Luciferase Analysis

HeLa and NIH 3T3 cells were cultured as described previously [33]. Cells were transfected with various plasmids using the Lipofectamine 3000 transfection reagent (Invitrogen). Briefly, HeLa cells were transfected with 400 ng of empty pcDNA3.1, 400 ng of wild-type KLF15-pcDNA3.1, 400 ng of Lys229*-mutant KLF15-pcDNA3.1, 200 ng of empty pcDNA3.1 plus 200 ng of wild-type KLF15-pcDNA3.1, or 200 ng of Lys229*-mutant KLF15-pcDNA3.1 plus 200 ng of wild-type KLF15-pcDNA3.1, in combination with 800 ng of KChIP2-luc and 40 ng of pGL4.75 (Promega). To analyze the inhibitory effect of KLF15 on CTGF, NIH 3T3 cells were transfected with 200 ng of empty pcDNA3.1, 200 ng of wild-type KLF15-pcDNA3.1, 200 ng of Lys229*-mutant KLF15-pcDNA3.1, 200 ng of TGFB1-pcDNA3.1, 200 ng of wild-type KLF15-pcDNA3.1 plus 200 ng of TGFB1-pcDNA3.1, or 200 ng of Lys229*-mutant KLF15-pcDNA3.1 plus 200 ng of TGFB1-pcDNA3.1, together with 800 ng of CTGF-luc and 20 ng of pGL4.75. The plasmid pGL4.75 expressing renilla luciferase was used as an internal control to normalize transfection efficiency. Cells were collected 36 h after transfection, and the luciferase activity of cellular lysates was assayed on a GloMax^®^ 96 Microplate Luminometer (Promega), with a Dual-Luciferase Reporter Assay System (Promega). The activity of the promoter was shown as the fold activation of firefly luciferase relative to renilla luciferase. Three independent transfection experiments were conducted in triplicate for each plasmid.

### 2.5. Statistical Analysis

Data for promoter activity were expressed as mean values ± standard deviation (SD) of three independent transfection experiments. Student’s unpaired *t* test was used for comparison between two groups, and analysis of variance with post hoc Fisher’s test was applied to comparisons between more than two groups. A two-tailed *p* value < 0.05 was considered statistically significant.

## 3. Results

### 3.1. Baseline Clinical Characteristics of the Study Subjects

We identified a four-generation AF family (Figure 1A), with 33 living family members (15 males and 18 females; aged from 12 years to 88 years). All affected family members had electrocardiogram-documented AF; while the unaffected family members had neither history of AF nor symptoms of AF, with normal electrocardiograms. None had structural heart disease or abnormalities in other organ systems. Genetic analysis of the pedigree showed that AF was transmitted in an autosomal dominant mode with complete penetrance. The index patient (II-3), a 66-year-old male with 34 years of personal AF history, was referred to Shanghai Chest Hospital because of syncope, and underwent catheter-based radiofrequency ablation of AF. The left atrial voltage maps of patient II-3 during circumferential pulmonary vein isolation showed extensive low-voltage areas in the left atrial anterior and posterior walls as well as atrium–pulmonary vein junctions, which could point to structural remodeling as a cause of AF. Notably, the proband’s father (I-1) and elder brother (II-1) died of stroke and ventricular tachycardias at the ages of 65 years and 63 years, respectively. Additionally, all affected living family members also had electrocardiogram-documented premature ventricular contraction, and four of them (II-3, III-1, III-6 and III-12) had electrocardiogram-documented non-sustained ventricular tachycardia. Furthermore, two affected living family members (II-8 and III-1) had hypertrophic cardiomyopathy (HCM) characterized by progressive left ventricle hypertrophy, though their echocardiographic images were normal at initial diagnosis of AF. The clinical characteristics of the affected family members are provided in Table 1. Additionally, there was no statistical difference in the echocardiographic or electrocardiographic parameters (as shown in Table 1) between unaffected family members (*n* = 24) and affected living family members (*n* = 9), except for the QRS interval, which was shorter in unaffected family members than in affected living family members (90.15 ± 7.16 ms vs. 98.00 ± 9.68 ms: *t* = 2.5462, *p* = 0.0161).

### 3.2. Identification of a New KLF15 Variation

WES was performed on the proband (II-3) and his affected son (III-7) and daughter (III-12), as well as his unaffected wife (II-4) and unaffected son (III-9) (Figure 1A), yielding a mean of 23 Gb of sequence data per sample, with an average of 97% of sequences mapped to the human reference genome (hg19) and 74% mapped to the target sequences. The mean sequencing depth was ~220×. An average of 17,128 exonic variants (range 16,094–18,528) per individual passed inheritance model filtering, of which 12 heterozygous missense, nonsense and splice site variants passed ANNOVAR filtering, shared by the three affected family members. Among the 12 candidate pathogenic variants, only the pathogenic variant chr3:126066633A>T (GRCh37.p13: GenBank accession no. NC_000003.11), equivalent to chr3:126293291A>T (GRCh38.p13: GenBank accession no. NC_000003.12) or NM_014079.4: c.685A>T; p.(Lys229*) in the KLF15 gene, was verified by Sanger sequencing with the primers shown in Table 2, and co-segregated with AF in the whole family. The electropherograms illustrating the heterozygous KLF15 mutation and its homozygous wild-type control sequence are presented in Figure 1B. The schematic diagrams depicting functionally important structural domains of wild-type and mutant KLF15 proteins are exhibited in Figure 1C. The mutation was neither observed in 600 control chromosomes nor reported in the ExAC, gnomAD or dbSNP databases, which were searched again on 6 January 2021, indicating that it is a novel mutation.

### 3.3. Functional Loss of the Mutant KLF15 Protein

**Figure 2:**
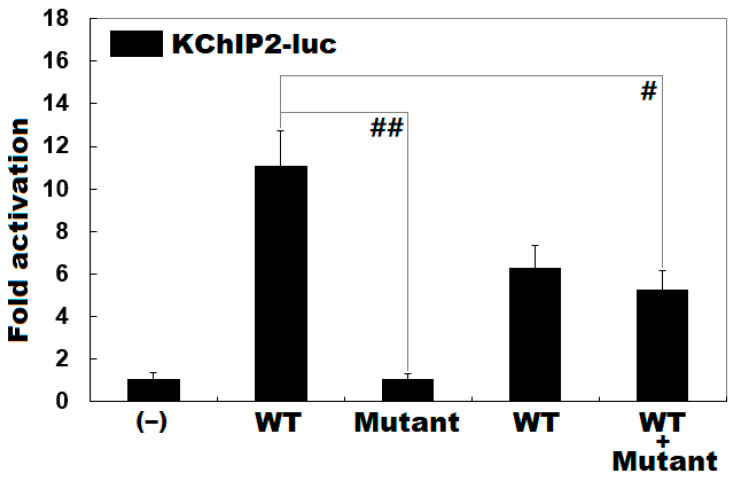
Functional loss of KLF15 caused by the mutation. In cultured HeLa cells, activation of the *KChIP2* promoter-driven luciferase by wild-type (WT) or Lys229*-mutant KLF15 (Mutant), alone or in combination, showed that the mutant had no transcriptional activity. Experiments were done in triplicate, and the results are expressed as mean values ± SD. ^##^ and ^#^ indicate *p* < 0.001 and *p* < 0.01, respectively, compared with the same amount of homozygous WT.

As shown in Figure 2, wild-type KLF15 (WT) strongly transactivated the transcription of the KChIP2 promoter, with a ~10-fold increase in reporter activity relative to basal level, blank control (‒); while Lys229*-mutant KLF15 (Mutant) did not transactivate the KChIP2 promoter, with no fold increase in reporter activity compared with blank control (WT vs. Mutant: *t* = 10.2154, *p* = 0.00052). In the heterozygous condition with an equimolar amount of WT and Mutant co-expressed, the induced transactivation of the KChIP2 promoter was reduced by ~50% (WT vs. WT + Mutant: *t* = 5.29602, *p* = 0.00610).

### 3.4. Diminished Inhibitory Effect of KLF15 on CTGF by the Mutation

**Figure 3:**
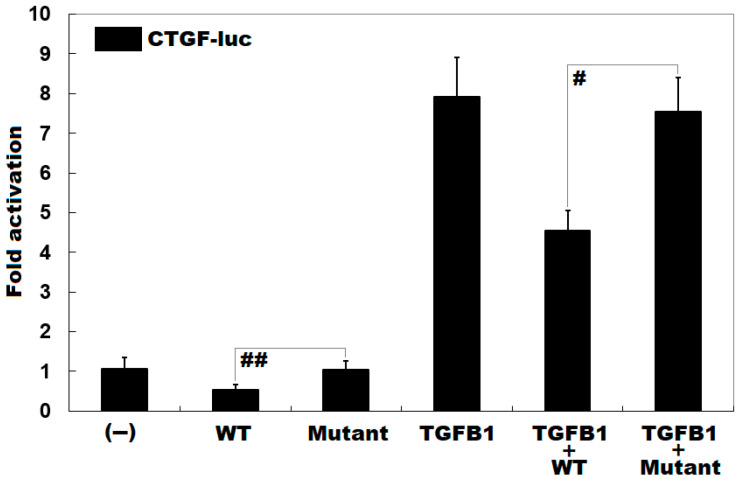
Diminished inhibitory effect of KLF15 on CTGF resulting from the mutation. In cultivated NIH 3T3 cells, activation of the *CTGF* promoter-driven luciferase by wild-type (WT) or Lys229*-mutant KLF15 (Mutant), in the absence or presence of TGFB1, showed that the inhibitory effect of the mutant on CTGF was significantly decreased. Experiments were carried out in triplicate, and the data were given as mean values ± SD. ^##^ and ^#^ indicate *p* < 0.05 and *p* < 0.01, respectively, in comparison with their wild-type counterparts.

As shown in Figure 3, the mutation decreased the inhibitory effect of KLF15 on the transcriptional activity of CTGF promoter by ~47% under basal conditions (WT vs. Mutant: *t* = ‒3.05806, *p* = 0.03773); while in the presence of TGFB1, the mutation diminished the inhibitory effect of KLF15 on the CTGF promoter by ~40% (WT + TGFB1 vs. Mutant + TGFB1: *t* = ‒5.12823, *p* = 0.006685).

## 4. Discussion

In the current investigation, a novel heterozygous mutation of c.685A>T (p.Lys229*) in the KLF15 gene was discovered in a family affected with AF as well as ventricular arrhythmias and hypertrophic cardiomyopathy by WES and bioinformatic analysis. The mutation was validated by Sanger sequencing and co-segregated with AF in the whole family with complete penetrance. The mutation was neither detected in 600 reference chromosomes nor reported in the ExAC, gnomAD or dbSNP databases. Biological assays revealed that the mutant KLF15 protein failed to transcriptionally activate the KChIP2 promoter. Furthermore, the mutation significantly reduced the inhibitory effect of KLF15 on the CTGF promoter, either under basic conditions or in the presence of TGFB1. Hence, this KLF15 variant may predispose to AF as well as ventricular arrhythmias and hypertrophic cardiomyopathy, but there may be a number of compensating mechanisms that the authors have not investigated.

In humans, KLF15 maps to chromosome 3q21.3, coding for a transcription factor protein consisting of 416 amino acids. Previous research has demonstrated that KLF15 functions as a transcriptional activator of several important target genes expressed in the heart, including KChIP2, ACSS2, GLUT4, PDK4 and FATP1 [38,40,41,42]. There is a consensus that the co-assembly of the pore-forming α subunit Kv4.3 (encoded by KCND3) with the regulating β subunit KChIP2 forms transient outward potassium current (Ito) channels, generating Ito responsible for the early repolarization of cardiac action potential [43]. As a critical modulatory subunit required for generating Ito, KChIP2 deficiency is sufficient to create conditions for arrhythmogenesis, including a complete absence of Ito and a pronounced increase in action potential duration, hence conferring an enhanced susceptibility to arrhythmias [44]. Moreover, as an upstream regulator of KChIP2, KLF15 transcriptionally controls rhythmic expression of KChIP2, and either deficiency or excess of KLF15 leads to loss of rhythmic QT variation, abnormal repolarization and increased vulnerability to arrhythmias [38]. Therefore, contrary to KCND3 gain-of-function mutation [45], KLF15 loss-of-function mutation identified in this study contributes to AF probably by delaying repolarization and prolonging the effective refractory period, which generates a matrix with increased trigger activity eliciting AF [18]. Additionally, abnormalities in cardiac energy metabolism have been associated with the initiation and persistence of AF [46,47]. Given that GLUT4, ACSS2, PDK4 and FATP1 play crucial roles in glucose and fatty acid metabolism [40,41,42], KLF15 loss-of-function mutation may also predispose to AF by disturbing myocardial energy metabolism.

Recently, atrial fibrosis has been demonstrated to be an important pathological contributor to AF occurrence and persistence, mainly by disrupting atrial electric conduction [48]. Atrial fibrosis is implicated with multiple profibrotic signaling molecules, encompassing CTGF, TGFB1, angiotensin-II and platelet-derived growth factor [49]. These profibrotic signaling molecules are interactive and incorporate positive feedback. Specifically, CTGF is up-regulated by TGFB1, which in turn is activated by angiotensin-II. Then fibroblasts that have been stimulated by CTGF, TGFB1, platelet-derived growth factor and angiotensin-II themselves synthesize and release profibrotic molecules, including TGFB1, CTGF and angiotensin [48]. Interestingly, there is convincing evidence demonstrating that KLF15 negatively regulates the expressions of CTGF and TGFB1, two key mediators of myocardial fibrosis, and can ameliorate or even reverse cardiac fibrosis and improve heart function [49]. In neonatal rat ventricular fibroblasts, overexpression of KLF15 suppressed basal and TGFB1-induced CTGF expression. In contrast, hearts from Klf15-null mice were associated with elevated CTGF levels and increased cardiac collagen deposition in response to hemodynamic stress [49]. Hence, the KLF15 loss-of-function mutation discovered in the present study confers an enhanced susceptibility to AF probably by CTGF-induced atrial fibrosis.

Interestingly, ventricular arrhythmias, including ventricular premature beat and paroxysmal ventricular tachycardia, were seen in the described AF kindred. Similarly, in Klf15-null mice, a markedly increased vulnerability to ventricular tachycardia was observed [38]. Importantly, Klf15-transgenic mice presented with spontaneous ventricular tachycardia, and succumbed to about 35% mortality by 4 months of age [38]. These results underscore the need for long-term electrocardiographic monitoring for the mutation carriers due to an increased risk of life-threatening ventricular tachyarrhythmias that predispose to sudden cardiac death. Obviously, genetic testing will enable physicians to facilitate precise diagnosis, risk stratification and early prevention of malicious arrhythmias, hence mitigating the risk of sudden cardiac death.

It has been shown that KLF15 is an important negative regulator of cardiac hypertrophy, and loss or repression of KLF15 leads to left ventricle hypertrophy, due to lack of inhibition of pro-hypertrophic transcription factors and stimulation of trophic and fibrotic signaling pathways [50]. Importantly, single nucleotide polymorphisms in KLF15 have been associated with cardiac hypertrophy in patients with diabetes mellitus [51], and in experimental chronic kidney disease, ventricular hypertrophy is associated with reduced expression of cardiac KLF15 [52]. In the present study, two AF patients in this family developed left ventricle hypertrophy, highlighting the need for regular echocardiographic evaluation of cardiac structure and function for the mutation carriers to make an early diagnosis of left ventricle hypertrophy, a common harbinger of heart failure and sudden cardiac death. However, we did not have a standard genetic investigation for the cause of HCM but only performed Sanger sequencing for the variant of interest. Hence, we could not rule out the possibility that another relevant variant contributed to HCM in this family. Genome-wide sequencing analysis would help to clarify if there is another relevant variant in this family that could explain HCM.

## 5. Conclusions

The current study identifies KLF15 as a causative gene contributing to AF as well as ventricular arrhythmias and hypertrophic cardiomyopathy, which provides novel insight into the molecular pathogenesis of cardiac arrhythmias and cardiomyopathy, with potential implications for genetic counseling and individualized prophylaxis for cardiac arrhythmias and cardiomyopathy in a subset of patients.

## Figures and Tables

**Table 1:** Clinical characteristics of pedigree members affected by atrial fibrillation.

Subject Information				Phenotype	Electrocardiogram			Echocardiogram					
Identity (Family 1)	Gender	Age at Study Enrollment (Years)	Age at Initial Diagnosis of AF (Years)	AF (Classification)	Heart Rate (Beats/min)	QRS Interval (ms)	QTc	LAD (mm)	LVEF (%)	IVSD (mm)	LVPWD (mm)	LVEDD (mm)	LVESD (mm)
I-1	M	65 ⁎	40	Permanent	–	–	–	–	–	–	–	–	–
II-1	M	63 ⁎	43	Permanent	–	–	–	–	–	–	–	–	–
II-3	M	66	32	Permanent	63	109	439	39	58	11	11	54	34
II-8	F	61	34	Permanent	69	105	461	37	65	22	15	55	36
III-1	M	45	26	Persistent	65	92	417	34	60	18	13	51	32
III-6	F	41	37	Paroxysmal	110	93	433	31	64	10	10	44	27
III-7	M	43	28	Persistent	91	83	447	35	62	11	10	48	31
III-12	F	39	35	Persistent	85	95	448	33	68	10	9	46	25
IV-1	M	22	18	Paroxysmal	106	90	416	30	63	9	8	43	23
IV-5	F	19	19	Paroxysmal	83	112	420	28	66	9	7	40	21
IV-7	M	16	16	Paroxysmal	91	103	441	28	62	8	7	38	21

AF—atrial fibrillation; F—female; IVSD—interventricular septum diameter; LAD—left atrial diameter; LVEDD—left ventricular end-diastolic diameter; LVEF—left ventricular ejection fraction; LVESD—left ventricular end-systolic diameter; LVPWD—left ventricular posterior wall diameter; M—male; QTc—corrected QT interval. Note: ⁎, age at death.

**Table 2:** Primers to amplify the coding exons and splice donors/acceptors of the *KLF15* gene.

Coding Exon	Forward Primer (5′→3′)	Reverse Primer (5′→3′)	Amplicon (bp)
1-a	CTTCTGACCAGGCCTCCTGT	GTCCTTGCTGTTGCCCTCAG	558
1-b	AGCCTACCCTGGAGGAGATTGA	CCTGCTGCACACCCAAGTAAG	743
2	GGGGACCCTCCCCTAATCCT	GCGGGTTCGAGGCTCTAAGT	517

## Data Availability

The data presented in this study are contained within this article.
